# Glabridin Suppresses Macrophage Activation by Lipoteichoic Acid In Vitro: The Crucial Role of MAPKs-IL-1β-iNOS Axis Signals in Peritoneal and Alveolar Macrophages

**DOI:** 10.3390/biom15020174

**Published:** 2025-01-24

**Authors:** Shaw-Min Hou, Chun-Ming Yang, Wei-Chieh Huang, Ssu-Wei Cheng, Ting-Lin Yen, Chih-Wei Hsia, Cheng-Ying Hsieh, Chih-Hsuan Hsia

**Affiliations:** 1 Graduate Institute of Medical Sciences, College of Medicine, Taipei Medical University, Taipei 110, Taiwan; cgh05414@cgh.org.tw (S.-M.H.); dr.yang1101@gmail.com (C.-M.Y.); d119110003@tmu.edu.tw (W.-C.H.); 2 School of Medicine, College of Medicine, Fu Jen Catholic University, New Taipei City 242, Taiwan; 3 Department of Cardiovascular Center, Cathay General Hospital, Taipei 106, Taiwan; 4 Department of Neurology, Chi Mei Medical Center, Tainan 710, Taiwan; 5 Department of Pharmacy, Shin Kong Wu Ho-Su Memorial Hospital, Taipei 111, Taiwan; t005461@gmail.com; 6 Department of Medical Research, Cathay General Hospital, Taipei 106, Taiwan; d119096015@tmu.edu.tw; 7 Department of Pharmacology, School of Medicine, College of Medicine, Taipei Medical University, Taipei 110, Taiwan; 8 Department of Medical Research, Taipei Medical University Hospital, Taipei 110, Taiwan; d119106003@tmu.edu.tw; 9 Translational Medicine Center, Shin Kong Wu Ho-Su Memorial Hospital, Taipei 111, Taiwan

**Keywords:** glabridin, lipoteichoic acid, MAPK pathway, NF-κB, IL-1β, iNOS, macrophages

## Abstract

Inflammation, a fundamental response to infection and injury, involves interactions among immune cells and signaling molecules. Dysregulated inflammation contributes to diseases such as autoimmune disorders and cancer. Interleukin-1 beta (IL-1β), produced by macrophages in response to lipoteichoic acid (LTA) from Gram-positive bacteria, is a key inflammatory mediator. Glabridin (GBD), a bioactive compound from licorice root, exhibits anti-inflammatory properties. This study investigates GBD’s effects on LTA-induced proinflammatory signaling in RAW 264.7 murine macrophages and alveolar macrophages, MH-S, focusing on *IL-1β* expression and signaling pathways. Cell viability assays confirmed that 20 μM GBD was non-cytotoxic. Confocal microscopy and quantitative PCR showed that GBD significantly reduced IL-1β fluorescence intensity, mRNA, and protein levels. GBD also inhibited inducible nitric oxide synthase (*iNOS*) expression and nitric oxide (NO) production. Further analysis revealed that GBD suppressed NF-κB p65 nuclear translocation and selectively modulated MAPK pathway activation by reducing JNK and p38 MAPK phosphorylation without affecting ERK. Studies using specific inhibitors demonstrated that IL-1β production reduction was mechanistically linked to MAPK pathway inhibition. These findings highlight GBD’s potential as a therapeutic agent for inflammatory diseases through its ability to modulate critical inflammatory mediators and signaling pathways.

## 1. Introduction

The body’s defense mechanism initiates inflammation as a crucial biological reaction to diverse challenges, such as pathogenic invasions, cellular damage, or self-directed immune responses [1]. This intricate physiological process unfolds through a sophisticated interplay of various immune system components. Key players in this response include tissue-resident phagocytes, such as macrophages, circulating granulocytes, such as neutrophils, and adaptive immune cells, particularly lymphocytes [2]. When stimulated by inflammatory triggers, these sentinel cells orchestrate a complex sequence of events. They secrete a variety of signaling molecules, including pro-inflammatory mediators and chemoattractants, which serve to alert and summon additional immune cells to the affected area [3]. In addition to its role in combating pathogens and promoting tissue repair, chronic or dysregulated inflammation is implicated in the onset of numerous diseases, such as autoimmune disorders, cardiovascular diseases, and cancer [4].

In investigating inflammatory processes, researchers utilize murine macrophage cell lines to gain detailed insights into cellular mechanisms. Specifically, RAW 264.7 cells, derived from mouse peritoneal macrophages, offer a versatile model for investigating broad inflammatory signaling pathways and cellular responses. Complementing this approach, MH-S cells—a murine alveolar macrophage cell line originating from lung tissue—provide a specialized model for examining respiratory-specific immune mechanisms [5]. These MH-S cells are particularly valuable for studying alveolar macrophage responses, including their interactions with inflammatory stimuli and their role in pulmonary immune defense. Both cell lines facilitate detailed examinations of cytokine production, inflammatory mediator release, and cellular signaling cascades, particularly in response to stimuli like lipoteichoic acid (LTA). By utilizing these complementary cellular models, researchers can comprehensively dissect the molecular intricacies of inflammatory responses.

Within the complex network of inflammatory processes, interleukin-1 beta (IL-1β) stands out as a key orchestrator [6]. This potent pro-inflammatory molecule, predominantly synthesized by stimulated macrophages and various other immune cells, is released in response to pathogenic invasion or tissue damage [7]. IL-1β’s influence stems from its interaction with dedicated receptors on cell surfaces, triggering a cascade of intracellular events. This interaction activates crucial signaling pathways, notably NF-κB and MAPK, which are central to the inflammatory response [8]. The NF-κB signaling pathway involves the translocation of NF-κB dimers, such as p65/p50, to the nucleus, where they initiate the transcription of pro-inflammatory genes [9]. The MAPK pathway includes key kinases like JNK, ERK, and p38, which regulate various cellular activities, including gene expression, cell differentiation, and inflammatory responses [10]. The far-reaching effects of IL-1β encompass several critical aspects of inflammation, including the induction of fever, the guidance of white blood cells to affected areas, and the stimulation of additional inflammatory mediators [11]. Dysregulated IL-1β signaling is associated with the development of various inflammatory diseases, highlighting its significance as a therapeutic target. Understanding the molecular mechanisms underlying IL-1β-mediated inflammation is crucial for developing targeted therapies aimed at modulating inflammatory responses and treating associated diseases [12].

Lipoteichoic acid (LTA), an essential component of Gram-positive bacterial cell walls, influences *IL-1β* expression and activity during inflammation [13]. The interaction of LTA with Toll-like receptor 2 (TLR2) on the surface of immune cells, particularly macrophages and monocytes, initiates a cellular response cascade that sets in motion a series of intracellular events that culminate in the synthesis of IL-1β [14]. LTA specifically activates the TLR2 pathway, which is distinct from the TLR4 pathway, activated by lipopolysaccharide (LPS) from Gram-negative bacteria. While LPS models Gram-negative bacterial inflammation, LTA represents a distinct pathway associated with Gram-positive bacterial infections. Research has elucidated that the LTA-mediated release of IL-1β is orchestrated through the stimulation of key signaling pathways, notably the NF-κB and MAPK cascades [15,16]. Consequently, IL-1β enhances the inflammatory response triggered by LTA, playing a crucial role in defending the host against bacterial infections [16,17]. In this study, LTA was selected to specifically investigate the macrophage response to Gram-positive bacterial components, which differ from the LPS response associated with Gram-negative bacteria. The use of LTA as the stimulus provides a unique opportunity to study the anti-inflammatory effects of GBD in macrophage activation, emphasizing LTA’s distinct role in Gram-positive bacterial inflammation and its therapeutic implications. However, excessive or dysregulated IL-1β production in response to LTA can contribute to tissue damage and exacerbate inflammatory conditions [16,18]. Investigating how LTA regulates *IL-1β* expression and signaling is essential for understanding inflammatory diseases caused by Gram-positive bacteria, offering potential avenues for therapeutic development.

Natural compounds and herbal remedies have shown promising therapeutic potential in modulating inflammatory processes through diverse mechanisms [19,20]. Glabridin (GBD), a bioactive compound extracted from licorice root (*Glycyrrhiza glabra*), is known for its potent anti-inflammatory effects [21]. It suppresses the function of crucial inflammation-promoting enzymes, notably cyclooxygenase-2 (COX-2) and inducible nitric oxide synthase (iNOS) [22]. These enzymes play pivotal roles in generating inflammatory substances, with COX-2 facilitating prostaglandin synthesis and iNOS driving nitric oxide production [20]. Furthermore, GBD influences the biosynthesis and cellular release of diverse pro-inflammatory signaling molecules. This includes its impact on key mediators, such as tumor necrosis factor-alpha (TNF-α) and various members of the interleukin family. These actions collectively contribute to the suppression of inflammatory responses in various experimental models. Furthermore, GBD possesses antioxidant properties that help reduce oxidative stress, which is often associated with inflammation and tissue damage [23]. By scavenging free radicals and enhancing endogenous antioxidant defenses, GBD contributes to mitigating inflammation and protecting tissues from oxidative injury [24]. Studies have demonstrated the potential of GBD to alleviate symptoms of inflammatory conditions such as arthritis, dermatitis, and gastrointestinal inflammation [25]. However, its specific effects on lipoteichoic acid (LTA)-induced inflammation and the underlying mechanisms remain areas of ongoing investigation. Elucidating the multifaceted anti-inflammatory mechanisms of GBD may unlock new avenues for innovative treatment approaches in the management of inflammatory disorders.

## 2. Materials and Methods

### 2.1. Reagents

Sigma-Aldrich (St. Louis, MO, USA) provided GBD (≥98% purity), dimethyl sulfoxide (DMSO), phenylmethylsulfonyl fluoride (PMSF), sodium orthovanadate, sodium pyrophosphate, aprotinin, leupeptin, sodium fluoride (NaF), and bovine serum albumin (BSA). Cell Signaling Technology (Beverly, MA, USA) supplied the anti-NF-κB p65 monoclonal antibody (mAb). Antibodies against phosphorylated forms of p38 MAPK, JNK, and ERK were acquired from Affinity (Cincinnati, OH, USA). Bio-Rad Laboratories Inc. (Hercules, CA, USA) furnished the protein assay dye reagent concentrate. Invitrogen (Thermo Fisher Scientific, Waltham, MA, USA) provided the anti-α-tubulin mAb and Alexa Fluor^®^ 488 Dye. Anti-IL-1β and anti-lamin B1 were purchased from Proteintech. Amersham (Buckinghamshire, UK) provided several key reagents: Hybond-P polyvinylidene difluoride membranes, enhanced chemiluminescence Western blotting detection reagent, and horseradish peroxidase-conjugated secondary antibodies (donkey anti-rabbit immunoglobulin G (IgG) and sheep anti-mouse IgG).

LTA (Sigma-Aldrich, L2515) was dissolved in sterile double-distilled water (ddH_2_O) as per the manufacturer’s recommendations. The stock solution was aliquoted and stored at −20 °C. GBD was prepared in DMSO and stored at 4 °C. Before use, both stock solutions were diluted to the working concentration in cell culture medium.

### 2.2. Cell Culture and Viability Assessment

RAW 264.7 murine macrophages were propagated in Dulbecco’s Modified Eagle Medium (DMEM) and MH-S cells were cultured in RPMI1640 medium, both of them enriched with 10% fetal bovine serum (FBS) and 1% penicillin-streptomycin. The cells were kept at 37 °C in a moisture-saturated environment with 5% CO_2_. For experimental procedures, cells were plated in appropriate culture vessels and allowed to adhere overnight.

For viability studies, cells were seeded at 1 × 10^5^ cells per well. Cells were pre-treated with GBD at various concentrations (5–20 µM) or vehicle control (0.1% DMSO) for 20 min, followed by stimulation with LTA (10 µg/mL) or left unstimulated for 24 h. Cell viability was evaluated using the MTT assay. The viability index was computed using the formula: (absorbance of treated cells/absorbance of control cells) × 100%. Absorbance readings were taken at 570 nm using an MRX reader (Dynex Technologies, Chantilly, VA, USA).

### 2.3. Quantification of Nitric Oxide Synthesis

NO production in cell culture supernatants was assessed using the Griess assay in a microplate format. RAW 264.7 cells were pre-incubated with GBD (5–20 μM) or vehicle control (0.1% DMSO) for 30 min, followed by stimulation with LTA or left untreated for 24 h. The resulting culture media were harvested and combined in equal parts with Griess reagent (comprised of 1% sulphanilamide and 0.1% naphthalenediamine dissolved in 2.5% phosphoric acid). Absorbance measurements were performed at 540 nm using an MRX spectrophotometer (Dynex Technologies, Chantilly, VA, USA). A sodium nitrite standard curve was employed for quantification.

### 2.4. Confocal Microscopy: Analysis of Protein Localization and Fluorescence Intensity

RAW 264.7 macrophages were pre-treated with DMSO, GBD or specific inhibitors (SP600125, SB203580, and PD98059, each at 10 μM) for 30 min and subsequently stimulated with LTA for 24 h, were cultured on coverslips. Cells were then fixed using 4% (*v*/*v*) paraformaldehyde for 10 min. The fixed cells underwent permeabilization with 0.1% Triton X-100, followed by a 30 min incubation in a 5% BSA-PBS solution to minimize non-specific binding. The prepared cells were then incubated with target-specific primary antibodies for 24 h at 4 °C. After thorough PBS washing, the specimens were exposed to Alexa Fluor^®^ 488-conjugated goat anti-rabbit IgG secondary antibody for 1 h at room temperature. Following three additional PBS washes, the coverslips were mounted onto glass slides using Fluoroshield medium containing DAPI.

Cellular imaging was performed using a Leica TCS SP5 confocal microscope (Mannheim, Germany) with a 60× oil immersion objective. Fluorescence intensity was analyzed using ImageJ 1.47v software. For IL-1β and iNOS analysis, the mean fluorescence intensity (MFI) of the whole cell was quantified by selecting regions of interest (ROIs) around individual cells. For p65 nuclear translocation, nuclear ROIs were identified based on DAPI staining. The MFI within the selected ROIs was calculated, and background intensity was subtracted using measurements from cell-free regions. The MFI values were directly measured for each group without normalization, as the focus was on comparing the relative fluorescence intensities across experimental groups.

### 2.5. RNA Extraction and Quantitative PCR (qPCR)

After treatment, RAW 264.7 cells were harvested using standard protocols. Total RNA was extracted using a commercial kit (Macherey-Nagel, Düren, Germany). Reverse transcription was performed to synthesize cDNA. Gene expression analysis was performed via real-time PCR utilizing primers targeting *IL-1β* and *iNOS*. GAPDH served as the reference gene for normalization, ensuring accurate quantification. Primer sequences for all target and reference genes are listed in Table 1. Quantitative analysis of gene expression was performed using the (2^−∆∆Ct^) method.

### 2.6. Immunoblotting

RAW 264.7 macrophages and MH-S cells (8 × 10^5^ cells/mL) were cultured and pre-incubated with either DMSO or GBD for 30 min, followed by LTA stimulation or left untreated for 24 h. Cells were then lysed in 100 μL of buffer containing protease and phosphatase inhibitors (aprotinin 10 μg/mL, PMSF 1 mM, leupeptin 2 μg/mL, NaF 10 mM, sodium orthovanadate 1 mM, and sodium pyrophosphate 5 mM). After 1 h of incubation in the lysis buffer at 4 °C, samples were centrifuged at 12000 rpm for 30 min. The resulting supernatants were harvested for protein analysis. Protein concentrations were determined using the Bradford protein assay (Bio-Rad, Hercules, CA, USA). For electrophoresis, 50 μg of protein from each sample was separated on 12% SDS-PAGE gels. Target proteins were identified using specific primary antibodies (diluted 1:1000 in TBST) for 2 h at 4 °C. After washing with TBST, membranes were incubated with HRP-conjugated secondary antibodies (diluted 1:5000) for 1 h at room temperature. Protein band intensities were quantified using a video densitometer and Bio-profil Biolight software (Version V2000.01, Vilber Lourmat, Marne-la-Vallée, France). The relative expression of proteins of interest was calculated by normalizing to total protein content. Original western blots can be found at Supplementary Materials. 

### 2.7. Statistical Analysis

Experimental results are presented as mean values ± standard error of the mean (SEM), with each experiment conducted a minimum of four times. To evaluate the significance of differences between experimental groups, one-way analysis of variance (ANOVA) was employed, followed by post-hoc comparisons using the Student–Newman–Keuls test. Statistical significance was established at *p* < 0.05.

## 3. Results

### 3.1. GBD’s Effects on Macrophage Viability and Morphological Changes

**Figure 1:**
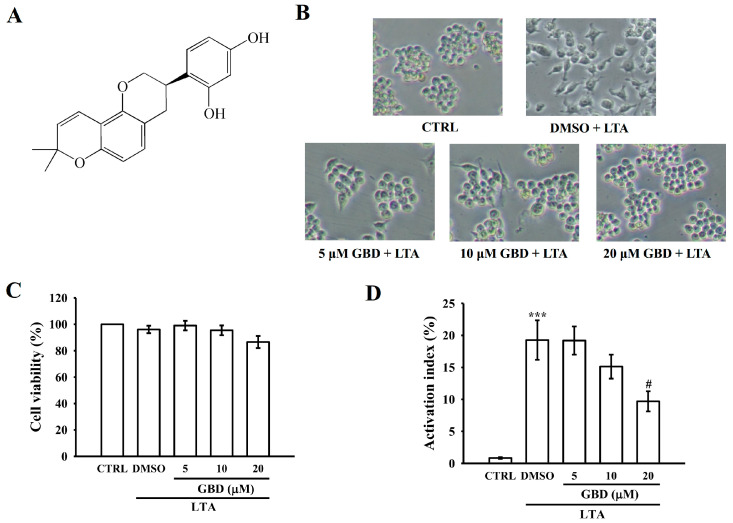
Impact of Glabridin (GBD) on morphology and availability in LTA-stimulated RAW 264.7 cells. (**A**) Chemical structure of GBD. (**B**) Representative phase-contrast microscopy images (200× magnification) showing cell morphology under different treatment conditions. Cells were exposed to control (CTRL), DMSO + LTA (10 μg/mL), or varying concentrations of 5, 10, 20 μM GBD + LTA for a 24 h period. (**C**) Cellular treatments were conducted as outlined in (**B**). (**D**) Activation indices of cells subjected to different treatment protocols. Cell treatments were performed as described in (**B**). Results are expressed as mean ± SEM (*n* = 4). *** *p* < 0.001 in comparison to CTRL; ^#^ *p* < 0.05 when compared to the DMSO + LTA group.

Figure 1A illustrates the chemical structure of GBD. This structural depiction serves as a reference for understanding the compound’s pharmacological significance in subsequent experimental analyses. GBD was evaluated for its effects on cell viability and activation in LTA-stimulated cells. Figure 1B demonstrates the cell morphology exposed to various treatments: control (CTRL), DMSO + LTA, and GBD + LTA at concentrations of 5, 10, and 20 μM, respectively. Cells treated with 20 μM GBD + LTA exhibited notable morphological differences compared to the DMSO + LTA group. Figure 1C depicts the viability of cells exposed to various treatments. Viability, as determined by the MTT assay, remained unaffected across all treatment groups, indicating no cytotoxic effects. Figure 1D presents the quantification of cell activation based on the morphological analysis shown in Figure 1B. The results demonstrate that 20 μM GBD significantly inhibited cell activation in LTA-stimulated cells. Therefore, the concentration of GBD is 20 μM, which was used in the subsequent experiments, based on its demonstrated effectiveness in previous studies, including the work of Shin et al. [21].

### 3.2. GBD Suppresses IL-1β Production in LTA-Activated Macrophages

**Figure 2:**
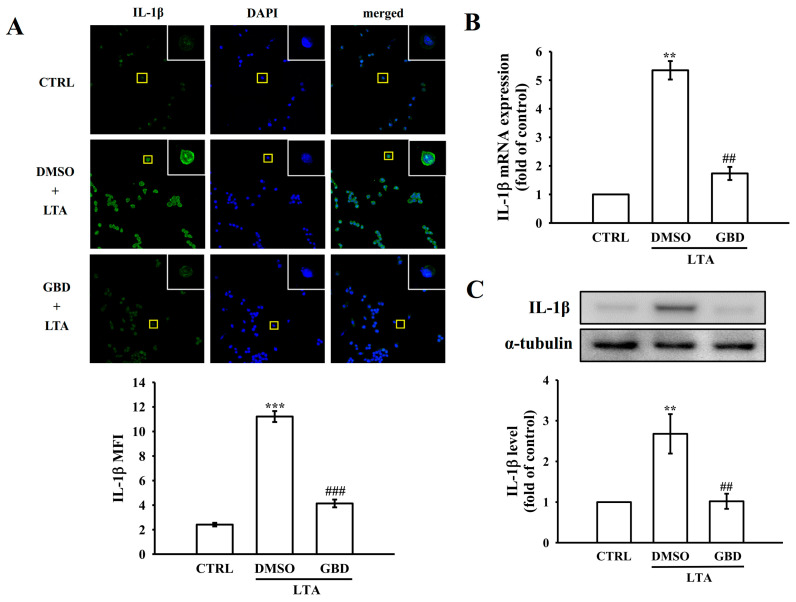
IL-1β production in response to LTA and GBD treatment. (**A**) Experimental groups: CTRL, DMSO + LTA, or GBD + LTA. The green shows IL-1β protein levels, and DAPI (blue) indicates cell nuclei. Insets show magnified views of individual cells (yellow squares). Lower panel: evaluation of IL-1β mean fluorescence intensity (MFI) in whole cells. Scale: 20 μm. (**B**) *IL-1β* mRNA levels were assessed via qRT-PCR and normalized to the reference genes GAPDH. (**C**) IL-1β protein analysis by Western blot. Upper panel: representative blot images of IL-1β and α-tubulin (loading control). Lower panel: quantification of IL-1β protein levels normalized to α-tubulin. For the mRNA and protein quantifications, the CTRL group was normalized to 1, and relative values for the treated groups were calculated accordingly. Error bars are not applicable to the CTRL group in panels (**B**,**C**) due to normalization to a fixed value of 1. Results are expressed as mean ± SEM (*n* = 4). ** *p* < 0.01 and *** *p* < 0.001 in comparison to CTRL; ^##^ *p* < 0.01 and ^###^ *p* < 0.001 when compared to the DMSO + LTA group.

The effect of GBD on interleukin-1 beta (IL-1β) production was examined by pre-treating macrophages with 20 μM GBD prior to LTA stimulation. Analysis using confocal laser scanning microscopy demonstrated that LTA activation significantly enhanced the fluorescence signal of IL-1β (depicted in green) compared to untreated control cells (Figure 2A). This enhancement was substantially diminished when cells were exposed to 20 μM GBD. The intensity of nuclear staining (shown in blue) remained stable across all experimental conditions (Figure 2A). Quantitative analysis of these fluorescence measurements is presented in the adjoining panels of Figure 2A.

*IL-1β* mRNA expression levels were significantly upregulated following LTA stimulation but were notably downregulated when macrophages were pre-treated with 20 μM GBD (Figure 2B). Complementary protein analysis using Western blotting confirmed an LTA-induced surge in IL-1β protein levels, which was substantially mitigated by GBD pre-treatment (Figure 2C). These observations indicate that GBD effectively curtails both the transcription and translation of IL-1β, underscoring its potential to modulate IL-1β-driven inflammatory cascades in macrophages.

### 3.3. GBD Modulates iNOS Protein Levels and NO Production in LTA-Activated Macrophages

**Figure 3:**
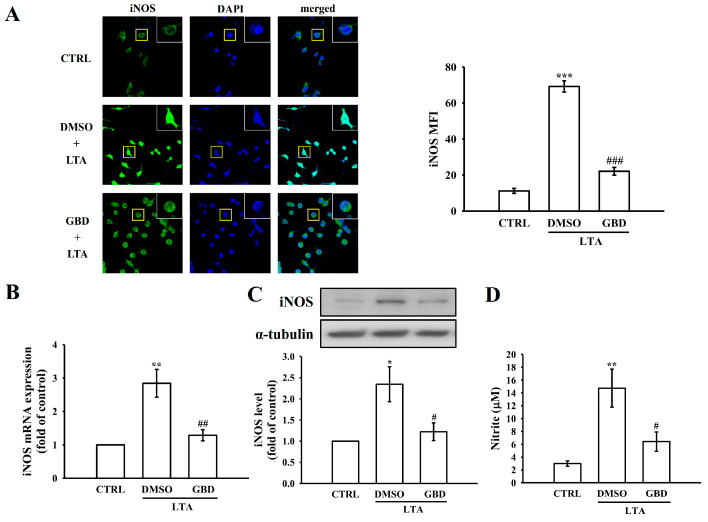
*iNOS* expression and NO production under LTA and GBD treatment. (**A**) Left: representative immunofluorescence images of iNOS protein levels. Experimental groups: CTRL, DMSO + LTA, or GBD + LTA. Insets show magnified views of individual cells (yellow squares). Right: evaluation of iNOS MFI in whole cells. Scale: 20 μm. (**B**) *iNOS* mRNA levels were assessed via qRT-PCR and normalized to the reference gene GAPDH. (**C**) iNOS protein analysis by Western blot. Upper panel: representative blot images of iNOS and α-tubulin (loading control). Lower panel: iNOS protein quantification, normalized to α-tubulin. (**D**) Nitrite production was measured by Griess assay. For the mRNA and protein quantifications, the CTRL group was normalized to 1, and relative values for the treated groups were calculated accordingly. Error bars are not applicable to the CTRL group in panels B and C due to normalization to a fixed value of 1. Results are expressed as mean ± SEM (*n* = 4). * *p* < 0.05, ** *p* < 0.01, and *** *p* < 0.001 in comparison to CTRL; ^#^ *p* < 0.05, ^##^ *p* < 0.01, and ^###^ *p* < 0.001 when compared to the DMSO + LTA group.

Given IL-1β’s role in stimulating *iNOS* expression and subsequent NO production, this study examined GBD’s potential impact on this cascade in macrophages exposed to LTA. Macrophages were pre-treated with GBD (20 μM) before LTA stimulation to assess its effects on iNOS protein levels. Confocal microscopy was used to visualize iNOS (green fluorescence) and cell nuclei (blue) in the macrophages. Control cells showed minimal iNOS detection. LTA exposure led to a notable increase in iNOS protein levels, as indicated by enhanced green fluorescence. In contrast, GBD pre-treatment substantially decreased iNOS fluorescence intensity in LTA-stimulated macrophages, indicating GBD’s inhibitory effect on iNOS upregulation (Figure 3A).

The results indicated that LTA stimulation significantly increased *iNOS* mRNA ex-pression compared to control cells. However, pre-treatment with 20 μM GBD significantly downregulated *iNOS* mRNA levels in LTA-stimulated macrophages, demonstrating that GBD inhibits *iNOS* transcription (Figure 3B). Following mRNA analysis, iNOS protein levels in macrophages were assessed via Western blot techniques. The data demonstrated a significant upregulation of iNOS protein levels in LTA-stimulated cells compared to the control group. Pretreatment with 20 μM GBD significantly attenuated the iNOS protein levels induced by LTA, consistent with the mRNA findings and indicating that GBD effectively inhibits iNOS protein production (Figure 3C).

To assess GBD’s effect on nitric oxide (NO) production, NO levels were quantified in the culture supernatants of treated macrophages. LTA stimulation significantly increased NO production compared to control cells. However, pre-treatment with 20 μM GBD markedly attenuated this increase, suggesting that GBD inhibits NO synthesis in response to LTA stimulation (Figure 3D).

### 3.4. GBD Inhibits NF-κB p65 Translocation in LTA-Stimulated Macrophages

**Figure 4:**
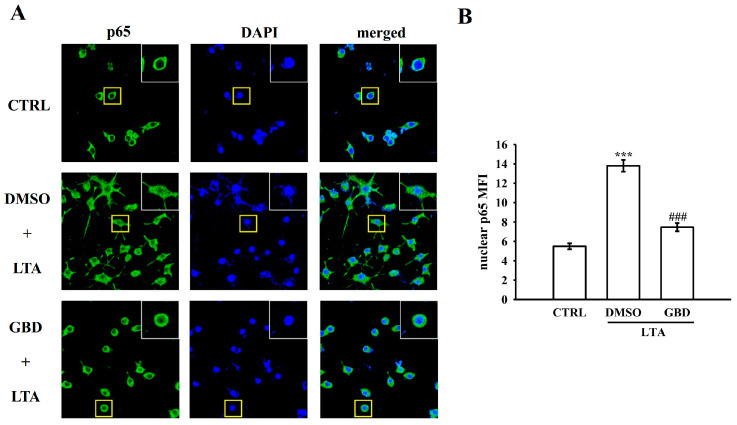
Evaluation of NF-κB p65 nuclear translocation in LTA and GBD-treated cells. (**A**) Representative immunofluorescence images showing nuclear localization of p65. Images highlight protein levels and nuclear translocation in CTRL, DMSO + LTA, and GBD + LTA groups. Yellow boxes show magnified views of individual cells. (**B**) Evaluation of nuclear p65 MFI from immunofluorescence images, representing nuclear translocation. Scale: 20 μm. Results are expressed as mean ± SEM (n = 4). *** *p* < 0.001 in comparison to CTRL; ^###^ *p* < 0.001 when compared to the DMSO + LTA group.

The impact of GBD on NF-κB p65 translocation was examined by pretreating macrophages with GBD (20 μM) before LTA stimulation. Immunofluorescence staining for p65 (green) and nuclear DAPI (blue) revealed that in the control group, p65 was predominantly localized in the cytoplasm. LTA stimulation resulted in an evident increase in nuclear p65, consistent with NF-κB activation. Notably, GBD pretreatment significantly attenuated this LTA-induced nuclear p65 fluorescence intensity (Figure 4A), indicative of reduced nuclear translocation of p65. Evaluation of nuclear fluorescence intensity of p65 confirmed a significant reduction in nuclear p65 levels in GBD-pretreated cells compared to the LTA-stimulated group (Figure 4B). These findings suggest that GBD may exert its anti-inflammatory effects, at least in part, by modulating NF-κB signaling in LTA-activated macrophages.

**Figure 5:**
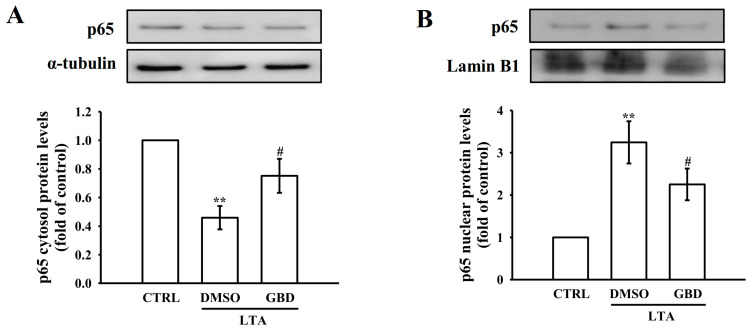
Cytosolic and nuclear distribution of NF-κB p65 following LTA and GBD treatment. (**A**) Western blot analysis of cytosolic p65 protein levels. Upper panel: representative blot images of p65 and α-tubulin (loading control). Lower panel: quantification of cytosolic p65 protein levels, normalized to α-tubulin. (**B**) Nuclear p65 protein levels were analyzed by Western blot. Upper panel: representative blot images of p65 and Lamin B1 (loading control). Lower panel: quantified nuclear p65 protein levels, normalized to Lamin B1. For all quantifications, the CTRL group was normalized to 1, and relative values for the treated groups were calculated accordingly. Error bars are not applicable to the CTRL group due to normalization to a fixed value of 1. Results presented as mean ± SEM (n = 4). ** *p* < 0.01 in comparison to CTRL; ^#^ *p* < 0.05 when compared to the DMSO + LTA group.

Western blot analysis was conducted to quantify p65 protein levels in cytosolic and nuclear fractions. The data revealed that LTA stimulation significantly increased nuclear p65 protein levels while decreasing cytosolic p65 protein levels compared to control cells. Pre-treatment with 20 μM GBD significantly reduced the nuclear p65 protein levels while increasing its cytosolic protein levels, aligning with the immunofluorescence results (Figure 5A,B). These findings suggest that GBD inhibits the nuclear translocation of p65 in response to LTA stimulation.

### 3.5. GBD Regulates MAPK Family Activation in LTA-Stimulated Macrophages

**Figure 6:**
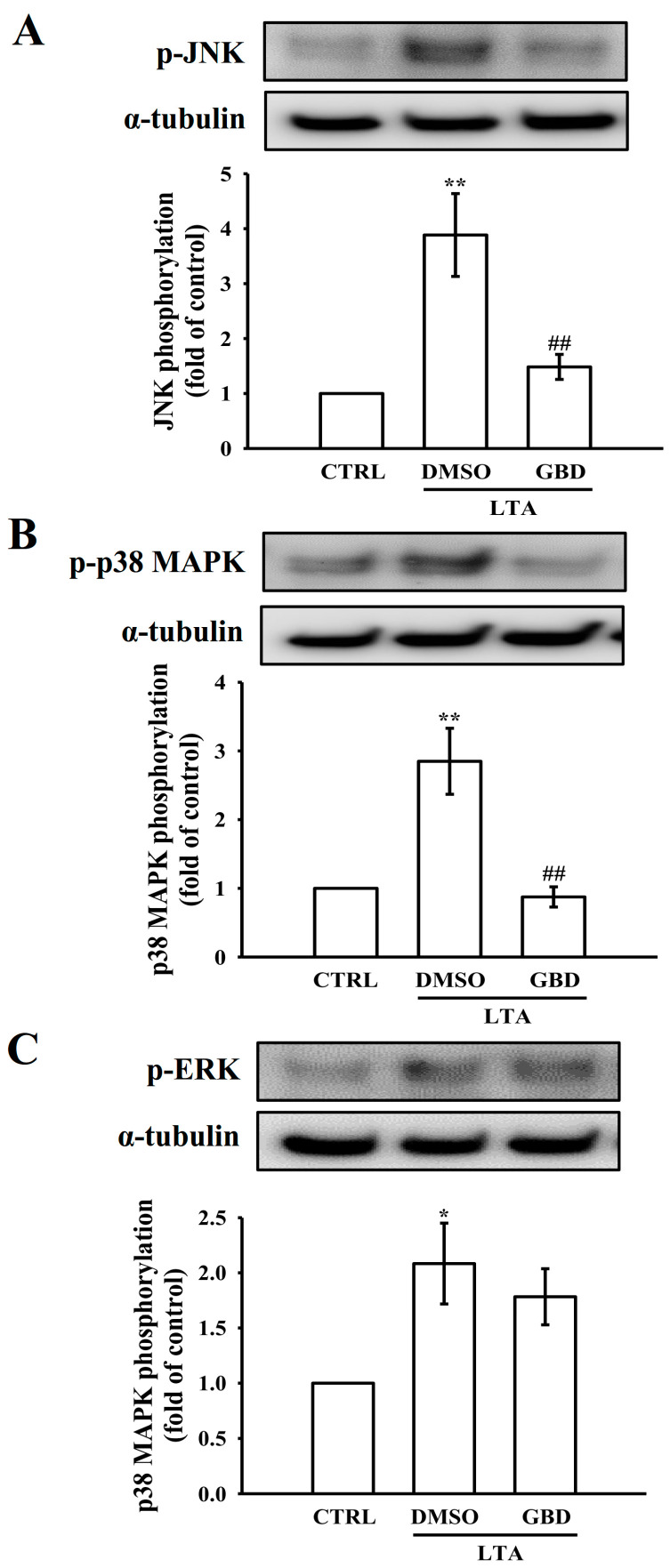
MAPK pathway phosphorylation in LTA and GBD-treated macrophages. (**A**–**C**) Experimental groups: CTRL, LTA + DMSO, or LTA + GBD. α-tubulin served as loading control. Bar graphs show fold changes in phosphorylation levels relative to control, normalized to α-tubulin. Western blot analysis and quantification of (**A**) p-JNK, (**B**) p-p38 MAPK, and (**C**) p-ERK levels. For all quantifications, the CTRL group was normalized to 1, and relative values for the treated groups were calculated accordingly. Error bars are not applicable to the CTRL group due to normalization to a fixed value of 1. Results are expressed as mean ± SEM (n = 4). * *p* < 0.05 and ** *p* < 0.01 in comparison to CTRL; ^##^ *p* < 0.01 when compared to the DMSO + LTA group.

MAPK pathways, including JNK, p38 MAPK, and ERK, play a significant role in regulating IL-1β protein levels. Activation of these pathways can lead to increased IL-1β production through various transcription factors and mRNA stability mechanisms. To elucidate GBD’s effect on MAPK family activation, the phosphorylation status of JNK, p38 MAPK, and ERK was examined in LTA-stimulated macrophages using Western blot analysis. In control macrophages, basal levels of phosphorylated JNK (p-JNK) were low. LTA stimulation significantly increased p-JNK levels, indicating activation of the JNK pathway. Pre-treatment with 20 μM GBD notably reduced the LTA-induced phosphorylation of JNK (Figure 6A). Like JNK, basal levels of phosphorylated p38 MAPK (p-p38) were low in control macrophages. LTA stimulation significantly elevated p-p38 levels, demonstrating p38 MAPK pathway activation. GBD pretreatment markedly attenuated LTA-induced p38 MAPK phosphorylation (Figure 6B). Phosphorylated ERK (p-ERK) levels were also low in control macrophages. LTA stimulation significantly increased p-ERK levels, reflecting ERK pathway activation. In contrast to its effects on JNK and p38 MAPK, GBD pre-treatment did not reduce the LTA-induced phosphorylation of ERK (Figure 6C). These findings indicate that GBD selectively modulates MAPK pathway activation in LTA-stimulated macrophages, effectively inhibiting JNK and p38 MAPK phosphorylation while not significantly affecting ERK phosphorylation.

### 3.6. GBD Targets JNK and p38 MAPK Pathways to Regulate LTA-Induced IL-1β Protein Levels

**Figure 7:**
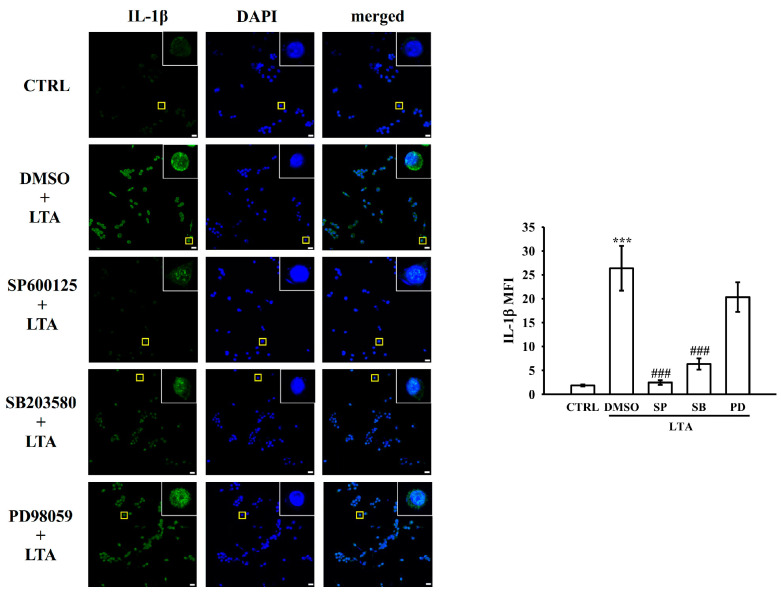
MAPK inhibitors on IL-1β protein levels in LTA-stimulated cells. Representative immunofluorescence images of IL-1β protein levels under different treatment conditions. Cells underwent 30-min pretreatment with vehicle (DMSO) or MAPK inhibitors (SP600125 for JNK, SB203580 for p38, PD98059 for ERK), then 24-h LTA exposure. Green: IL-1β protein levels; Blue: nuclei (DAPI-stained). Yellow boxes show magnified views of individual cells. Evaluation of IL-1β MFI in whole cells. Scale: 20 μm. Results are expressed as mean ± SEM (n = 4). *** *p* < 0.001 in comparison to CTRL; ^###^ *p* < 0.001 when compared to the DMSO + LTA group.

Immunofluorescence analysis revealed distinct patterns of IL-1β protein levels under various treatment conditions (Figure 7). Control cells showed minimal green fluorescence for IL-1β, indicating low basal protein levels. Treatment with DMSO + LTA significantly increased IL-1β fluorescence intensity (*p* < 0.001), suggesting LTA-induced IL-1β production. To further elucidate the role of MAPK pathways in LTA-induced IL-1β protein levels, researchers employed three specific inhibitors: SP600125 (JNK inhibitor), SB203580 (p38 MAPK inhibitor), and PD98059 (ERK inhibitor). Findings showed that pretreatment with either SP600125 or SB203580 markedly attenuated the LTA-induced enhancement of IL-1β fluorescence. In contrast, the ERK inhibitor PD98059 did not exhibit significant inhibitory effects. Quantitative analysis of mean fluorescence intensity corroborated these observations (Figure 7, right panel). These results suggest that LTA induces IL-1β protein levels through JNK and p38 MAPK-dependent pathways, while the ERK pathway may not be critically involved in this process. This discovery provides new insights into the molecular mechanisms underlying LTA-mediated inflammatory responses. Simultaneously, it identifies potential intervention targets for modulating IL-1β production in response to bacterial stimuli.

### 3.7. GBD’s Effects on LTA-Induced Activation of MH-S Cells

**Figure 8:**
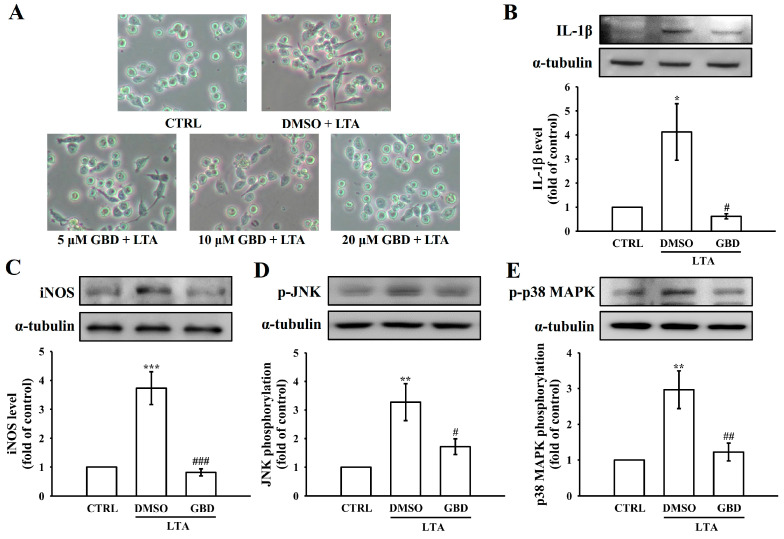
GBD suppresses LTA-induced inflammatory responses in MH-S alveolar macrophages. (**A**) Representative images showing the morphological changes in MH-S cells under different treatment conditions (200× magnification). Western blot analysis of (**B**) IL-1β, (**C**) iNOS, (**D**) p-JNK, and (**E**) p-p38 MAPK protein levels after LTA stimulation with or without GBD treatment. For all quantifications, the CTRL group was normalized to 1, and relative values for the treated groups were calculated accordingly. Error bars are not applicable to the CTRL group due to normalization to a fixed value of 1. Results are expressed as mean ± SEM (n = 4). * *p* < 0.05, ** *p* < 0.01, and *** *p* < 0.001 in comparison to CTRL; ^#^ *p* < 0.05, ^##^ *p* < 0.01, and ^###^ *p* < 0.001 when compared to the DMSO + LTA group.

To further evaluate the anti-inflammatory effects of GBD, additional experiments were conducted using the MH-S cells (mouse alveolar macrophages). As shown in Figure 8A, the control group (CTRL) exhibited a typical macrophage morphology with round, discrete cells. In contrast, the DMSO + LTA treatment group displayed an altered morphology, with the cells appearing more spread out and irregular in shape. Importantly, the 20 μM GBD + LTA treatment group exhibited a morphology that was more similar to the CTRL group compared to the DMSO + LTA condition, suggesting GBD may have modulated the LTA-induced morphological changes in these macrophages.

Consistent with the findings in RAW 264.7 cells, GBD treatment significantly inhibited LTA-induced activation of key inflammatory signaling pathways in MH-S cells. Specifically, GBD downregulated IL-1β protein levels (*p* < 0.05) and iNOS protein levels (*p* < 0.001) in MH-S cells exposed to LTA. Furthermore, GBD reduced phosphorylation of JNK (*p* < 0.05) and phosphorylation of p38 MAPK (*p* < 0.01) compared to the LTA-stimulated group (Figure 8B–E). These results in the MH-S alveolar macrophage model corroborate the anti-inflammatory properties of GBD observed in the RAW 264.7 cell line, reinforcing the compound’s ability to modulate the MAPKs-IL-1β-iNOS axis across different macrophage subtypes. The consistency of these findings strengthens the conclusion that GBD’s targeted suppression of key inflammatory mediators represents a promising therapeutic approach for managing inflammatory respiratory conditions.

## 4. Discussion

This study investigates GBD’s potent anti-inflammatory effects, particularly its ability to mitigate LTA-induced macrophage activation and inflammatory mediator production. Unlike the extensively studied LPS-induced inflammation, immune responses to Gram-positive bacterial components, particularly LTA, exhibit distinct characteristics and pose unique challenges. LTA plays a crucial role in infection processes and post-infection pathological developments by activating specific pattern recognition receptors distinct from those targeted by LPS. Previous studies have demonstrated LTA’s capacity to induce proinflammatory mediators in RAW 264.7 macrophages [16,26], making it an ideal model for studying anti-inflammatory interventions targeting Gram-positive bacterial infections. This focus on LTA-induced inflammation provides valuable insights into targeting inflammatory pathways unique to Gram-positive bacterial infections and underscores the therapeutic potential of GBD in these contexts.

In our experiments, DMSO was used as the control (CTRL) group to assess the baseline effects of the solvent on cell viability, gene transcription, and protein levels. As shown in Supplementary Figure S1, the results confirm that neither DMSO nor LTA alone significantly influenced cell viability, validating the use of DMSO as a control. While LTA alone did not affect cell viability, it is recognized as a potent pro-inflammatory stimulus in macrophages, typically inducing inflammatory responses in other contexts.

IL-1β has been extensively studied for its pivotal role in orchestrating inflammatory responses and contributing to the development of various inflammatory conditions [11,27,28]. Our research reveals that GBD significantly attenuates IL-1β secretion in macrophages exposed to LTA. Building on the work of Shin et al. [21] and Park et al. [29], which demonstrated the anti-inflammatory activity of GBD and its derivatives by targeting MAPKs and NF-κB pathways, our study extends these findings by focusing on LTA-induced macrophages. While their studies provided important insights into GBD’s mechanisms in LPS-induced inflammation, our work uniquely extends these findings to LTA-induced macrophages, offering new perspectives on GBD’s therapeutic potential against Gram-positive bacterial infections.

The present study differs from previous investigations in several key aspects. First, while Shin et al. [21] focused on LPS-induced inflammation and demonstrated GBD’s effects on TNF-α and IL-6 production, our study specifically examines the IL-1β-iNOS axis in LTA-stimulated conditions. Second, Park et al. [29] investigated synthetic GBD derivatives, whereas our work focuses on the native GBD molecule, providing essential insights into the natural compound’s efficacy. Additionally, our study uniquely demonstrates GBD’s selective modulation of MAPK pathways in LTA-stimulated cells, showing specific inhibition of JNK and p38 MAPK while leaving ERK phosphorylation unaffected.

Confocal microscopy analysis demonstrated a significant reduction in IL-1β fluorescence intensity in macrophages pre-treated with GBD compared to the LTA-stimulated group. This reduction was further supported by the downregulation of *IL-1β* mRNA and protein levels, as evidenced by qPCR and Western blot analysis, respectively. Furthermore, our investigation uncovered GBD’s modulatory effects on *iNOS* expression and protein level, and NO production in LTA-activated macrophages, both of which are intricately linked to IL-1β activity. iNOS, a key enzyme responsible for generating large quantities of NO during inflammation [30], can be influenced by IL-1β levels [20,21,24,31], highlighting the interconnected nature of these inflammatory mediators.

Understanding GBD’s impact on NF-κB and MAPK signaling pathways is crucial, given their central role in regulating *IL-1β* expression in LTA-stimulated macrophages. Our research revealed that GBD impedes the nuclear translocation and activation of NF-κB p65 in macrophages exposed to LTA. Through immunofluorescence and Western blot analyses, we confirmed GBD’s ability to reduce NF-κB p65 nuclear localization, thereby inhibiting its transcriptional activity and subsequent IL-1β production. These observations align with Chang et al.’s findings [32], which demonstrated GBD’s suppression of NF-κB activation in an atopic dermatitis mouse model, though in a different context.

Moreover, our study showed that GBD selectively modulated MAPK pathway activation in LTA-stimulated cells. Specifically, GBD inhibited the phosphorylation of JNK and p38 MAPK, while leaving ERK phosphorylation unaffected, which is consistent with LPS-stimulated macrophages [21]. This selective inhibition pattern suggests a more nuanced mechanism of action than previously understood. The differential effects on MAPK pathway components indicate that GBD’s anti-inflammatory activity involves specific targeting of distinct signaling cascades rather than general pathway suppression. Furthermore, our investigation into both peritoneal and alveolar macrophages provides a broader perspective on GBD’s effects across different macrophage populations, extending the therapeutic implications of these findings.

MH-S cells, a murine alveolar macrophage cell line, offer a unique and critical model for pulmonary immunological research. Unlike RAW 264.7 cells, which are derived from a murine macrophage-like cell line typically used for general inflammatory studies, MH-S cells more closely mimic the resident macrophages found in lung tissue. These cells are particularly valuable because they provide a more physiologically relevant platform for investigating inflammatory responses specific to the pulmonary environment [5,33]. RAW 264.7 cells, while widely used in initial screening and fundamental inflammatory research, represent a more generalized macrophage model that may not fully capture the nuanced immune characteristics of lung-specific macrophages. The comparative analysis between RAW 264.7 and MH-S cells in our study revealed consistent anti-inflammatory effects of GBD, strengthening the reliability of our findings. In both cell lines, GBD demonstrated a significant ability to suppress inflammatory responses, particularly through the modulation of the MAPKs-IL-1β-iNOS signaling axis. This consistency across different macrophage models suggests a robust and potentially generalizable mechanism of action for GBD in inflammatory processes. Clinically, these findings hold profound implications for managing inflammatory respiratory conditions. The ability of GBD to selectively inhibit inflammatory mediators in lung-specific macrophages suggests a promising therapeutic approach for diseases characterized by dysregulated inflammatory responses. Conditions such as chronic obstructive pulmonary disease (COPD), acute respiratory distress syndrome (ARDS), and potentially pulmonary fibrosis [34] could benefit from a targeted intervention that reduces inflammatory mediator production without completely suppressing immune function. The selective modulation of JNK and p38 MAPK pathways, coupled with the downregulation of IL-1β and iNOS, indicates a nuanced approach to managing inflammatory responses that could minimize tissue damage while maintaining essential immune surveillance.

While these results are promising, significant challenges remain in translating these in vitro observations into clinical applications. The current study provides a crucial foundation for understanding GBD’s anti-inflammatory mechanisms, but further research is essential. Future investigations should prioritize in-depth molecular characterization, in vivo validation, and exploration of GBD’s therapeutic potential, including studies on its efficacy, safety, and long-term effects across various inflammatory models and clinical contexts. Expanding the scope of inflammasome-related cytokines, including IL-1α and the extracellular secretion of IL-1β, will provide a more comprehensive understanding of GBD’s broader implications in inflammation.

## 5. Conclusions

**Figure 9:**
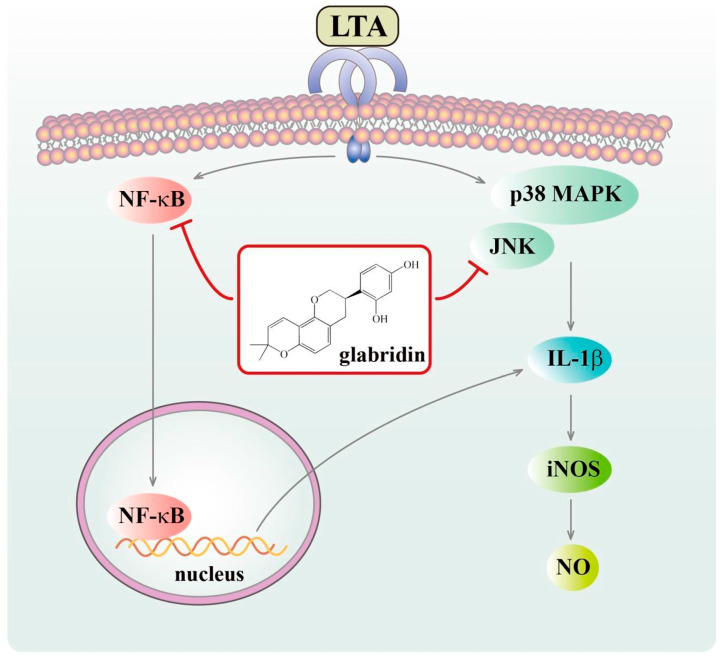
GBD’s inhibitory mechanism in LTA-activated macrophages. Schematic depicts LTA binding to its receptor, triggering NF-κB and JNK/p38 MAPK phosphorylation, leading to NF-κB p65 nuclear translocation. Activated JNK/p38 MAPK and translocated NF-κB p65 induce IL-1β, subsequently iNOS, culminating in NO production. GBD counteracts LTA-induced effects by suppressing NO generation, *IL-1β* and *iNOS* expression, JNK/p38 and NF-κB p65 phosphorylation, and NF-κB p65 nuclear translocation, thereby exhibiting anti-inflammatory properties.

This study enhances our understanding of GBD’s molecular mechanisms in mitigating LTA-induced inflammation. As illustrated in Figure 9, LTA triggers inflammatory responses through TLR2 receptor binding, leading to the activation of NF-κB and MAPK signaling pathways. GBD effectively interrupts this inflammatory cascade by inhibiting the phosphorylation of JNK/p38 MAPK and preventing NF-κB p65 nuclear translocation, thereby suppressing the production of inflammatory mediators IL-1β, iNOS, and NO. Our findings highlight GBD’s ability to interfere with multiple signaling cascades involved in IL-1β production, offering new insights into its anti-inflammatory properties. The demonstrated inhibitory effects of GBD on IL-1β protein levels, coupled with its modulation of iNOS protein levels and NO production, collectively underscore its potential as an anti-inflammatory agent. While these results are promising, further research is needed to fully elucidate GBD’s effects on other inflammatory pathways and in diverse cellular contexts. Future studies should explore GBD’s potential in various inflammatory models, investigate its long-term efficacy and safety as a therapeutic agent, and further delineate the molecular mechanisms underlying its anti-inflammatory actions. These efforts will contribute to evaluating GBD’s potential as a candidate for treating inflammatory diseases and may provide a foundation for developing novel anti-inflammatory strategies.

## Figures and Tables

**Table 1:** Primer sequences.

Gene	Primer Sequence
*IL-1β*	Forward 5′-CTC ATT GTG GCT GTG GAG AA-3′
	Reverse 5′-CAC ACA CCA GCA GGT TAT CA-3′
*iNOS*	Forward 5′-AGC CAA GCC CTC ACC TAC TT-3′
	Reverse 5′--GCC TCC AAT CTC TGC CTA TC-3′
*GAPDH*	Forward 5′-GAA CAT CAT CCC TGC ATC CA-3′
	Reverse 5′-GCC AGT GAG CTT CCC GTT CA-3′

## Data Availability

All data generated or analyzed during this study are included in this published article.

## Supplementary Materials

The following supporting information can be downloaded at: https://www.mdpi.com/article/10.3390/biom15020174/s1, Figure S1: Effects of DMSO and LTA alone on cell viability in RAW 264.7 and MH-S cells. File S1: Original western blots.
