# Supplementary material for: Glabridin Suppresses Macrophage Activation by Lipoteichoic Acid In Vitro: The Crucial Role of MAPKs-IL-1β-iNOS Axis Signals in Peritoneal and Alveolar Macrophages
Source: Biomolecules. 2025 Jan 24;15(2):174. doi: 10.3390/biom15020174 (PMC11853366; doi:10.3390/biom15020174)

Figure 2C IL-1β

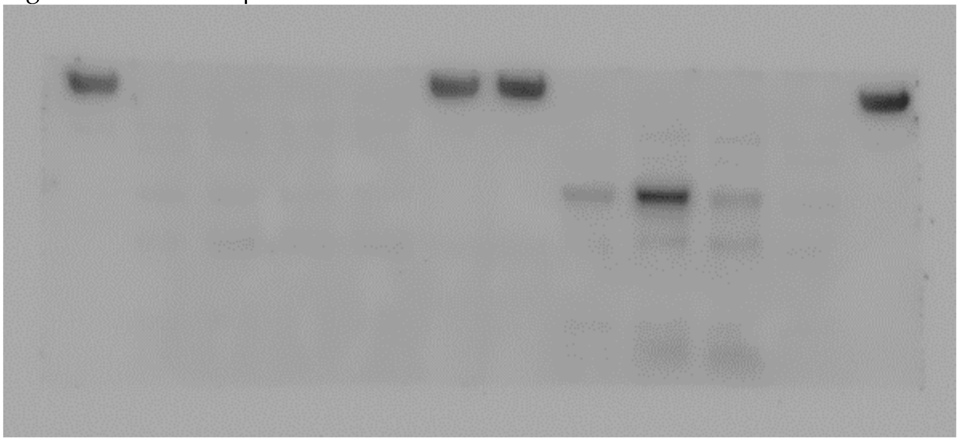

Figure 2C α-tubulin

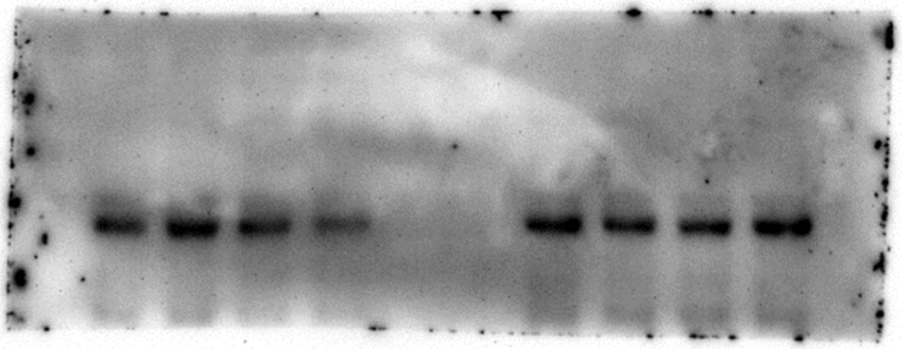

Figure 3C iNOS

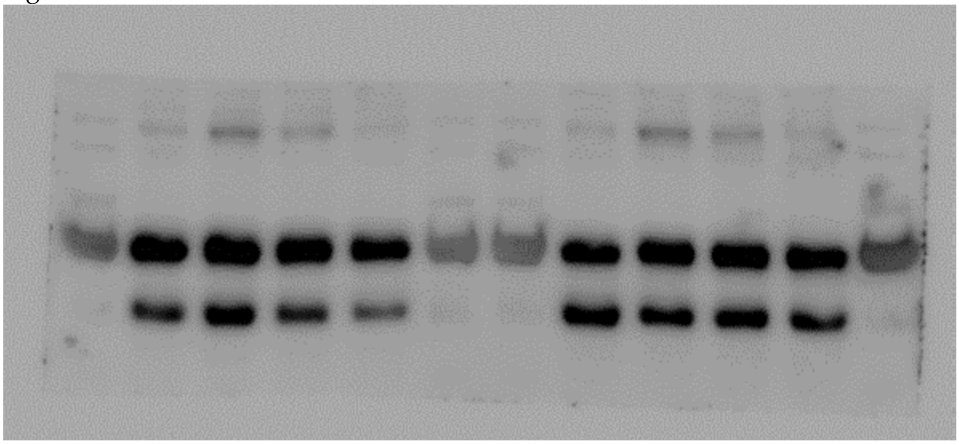

Figure 3C α-tubulin

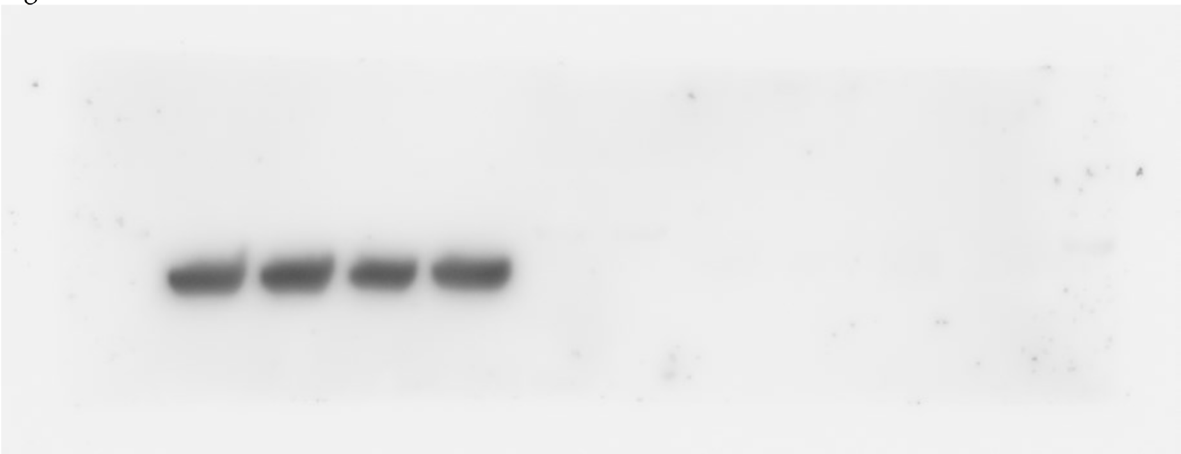

Figure 5A p65

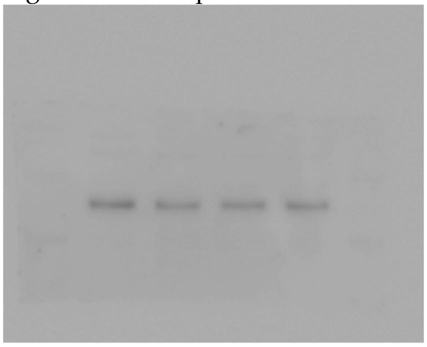

Figure 5A α-tubulin

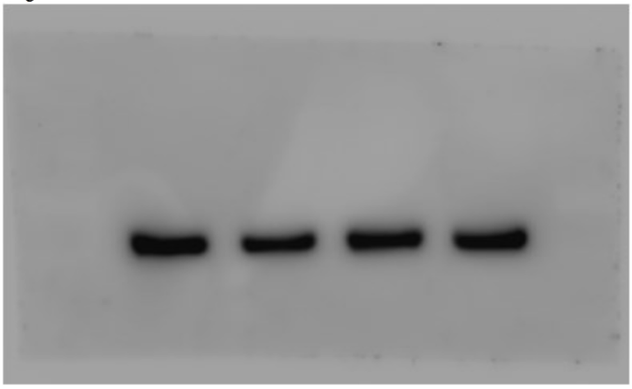

Figure 5B p65

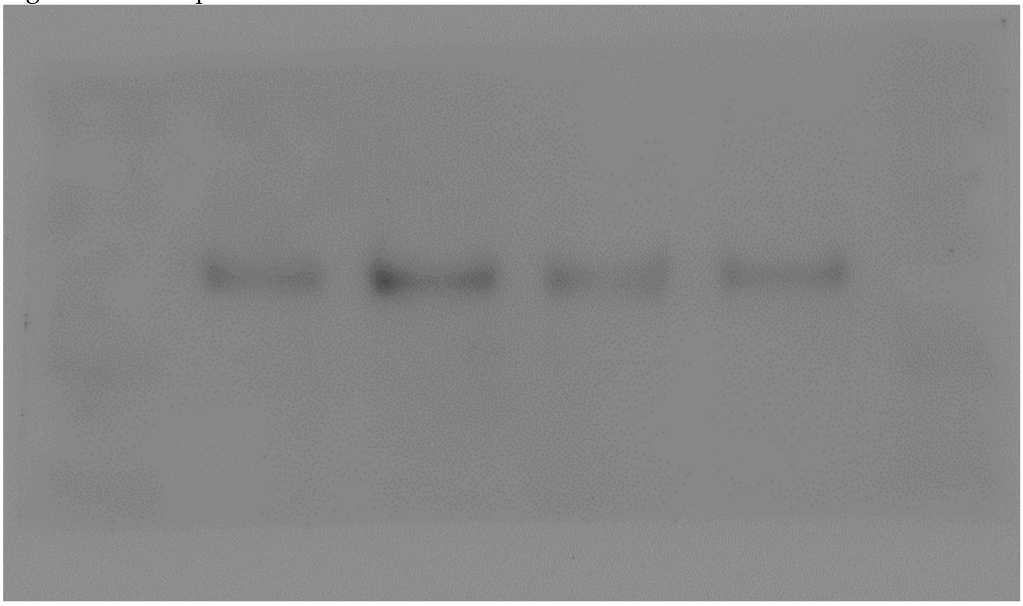

Figure 5B Lamin B1

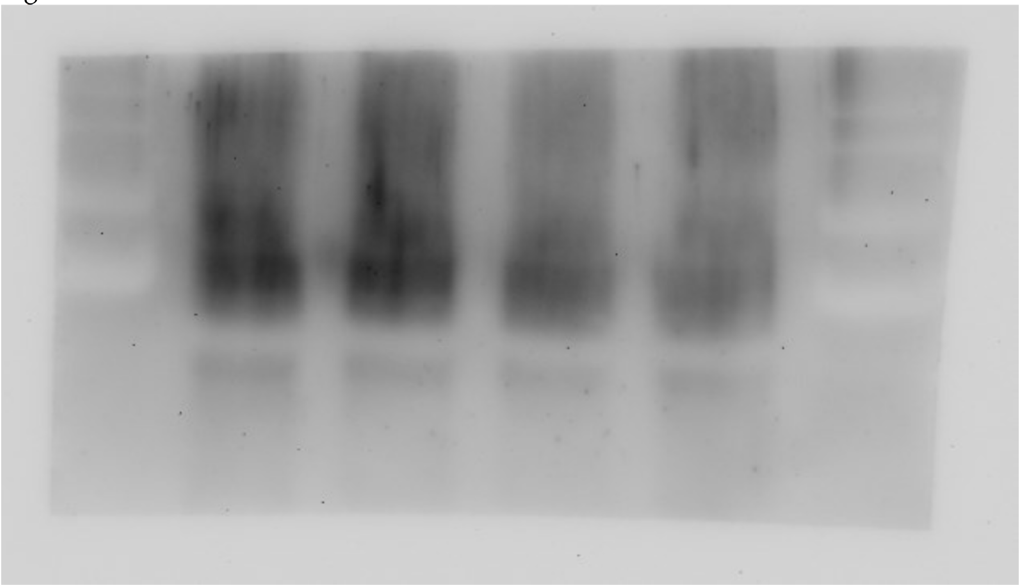

Figure 6A p-JNK

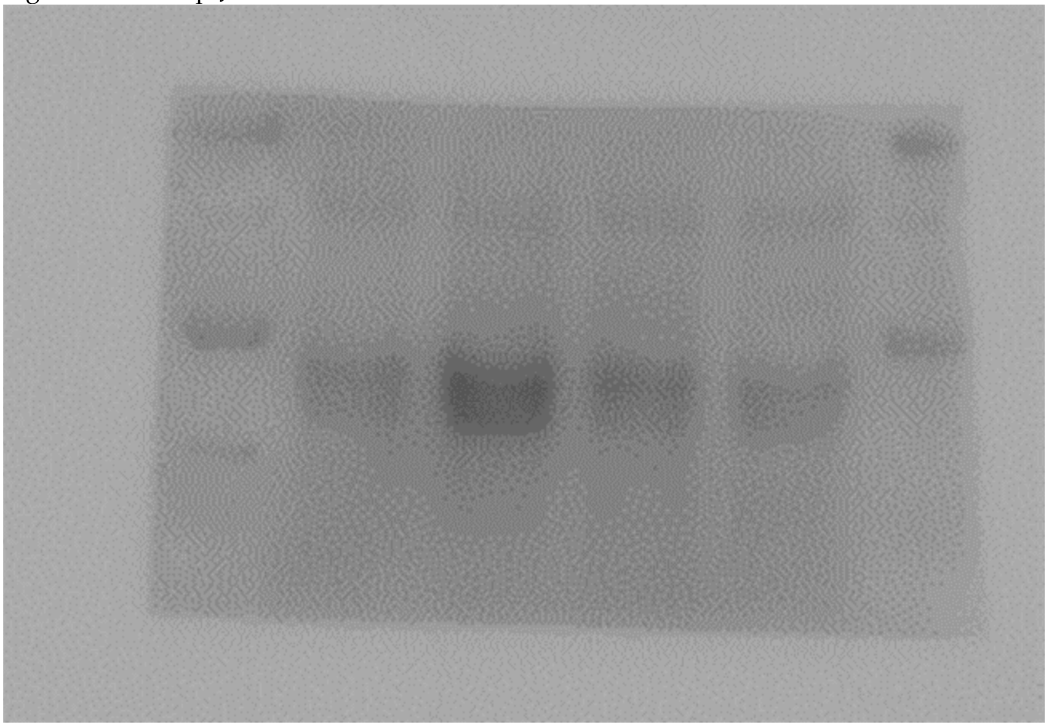

Figure 6A α-tubulin

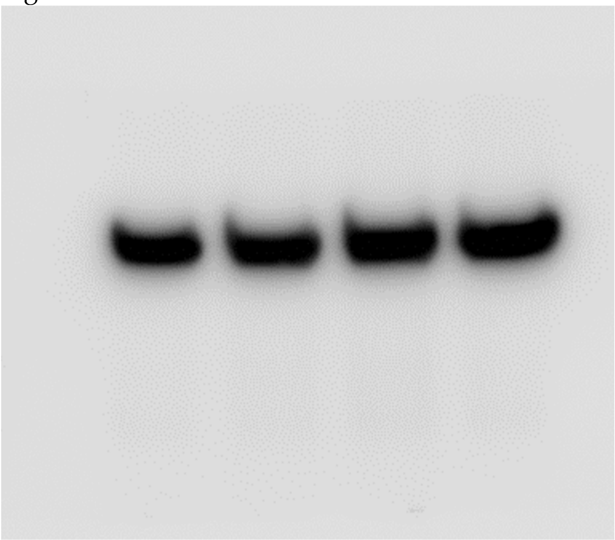

Figure 6B p-p38 MAPK

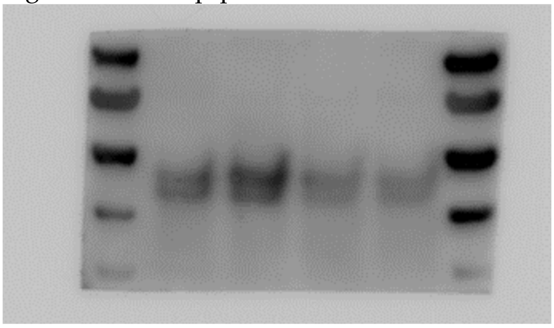

Figure 6B α-tubulin

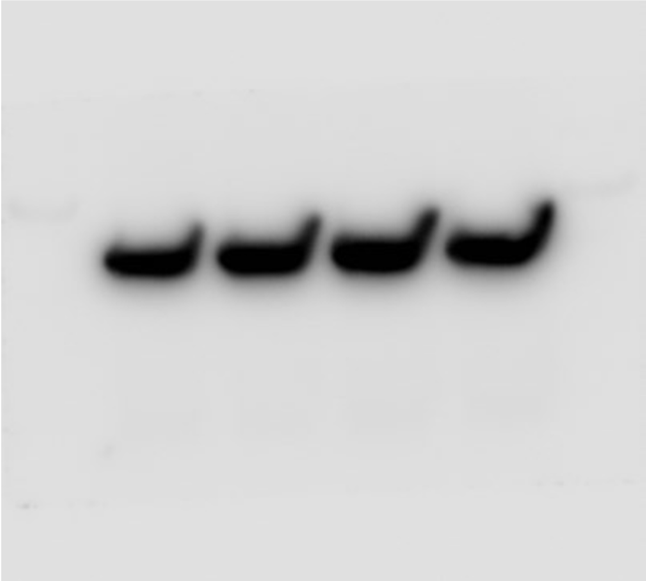

Figure 6C p-ERK

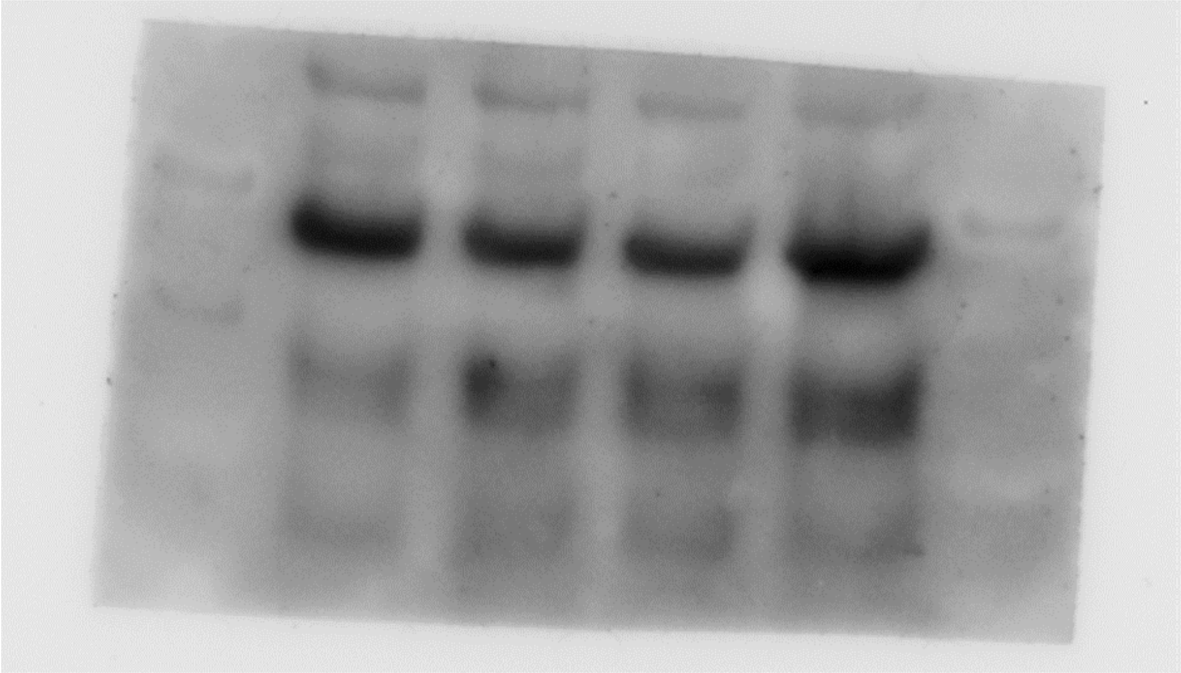

Figure 6C α-tubulin

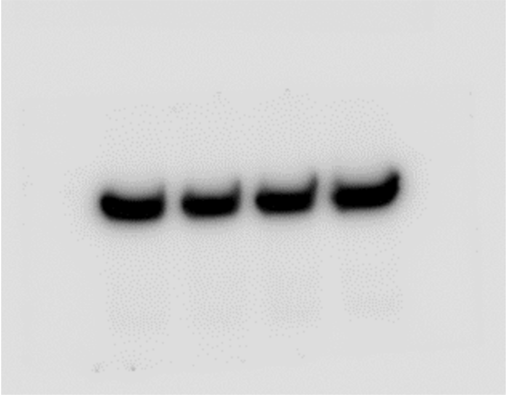

Figure 8B IL-1β

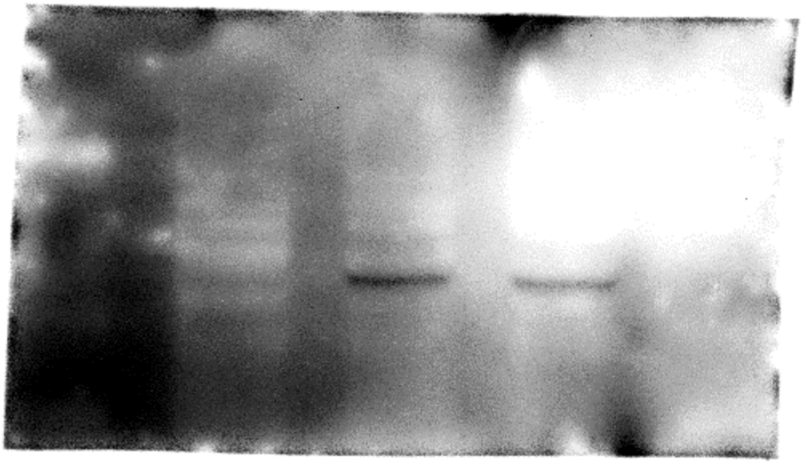

Figure 8B α-tubulin

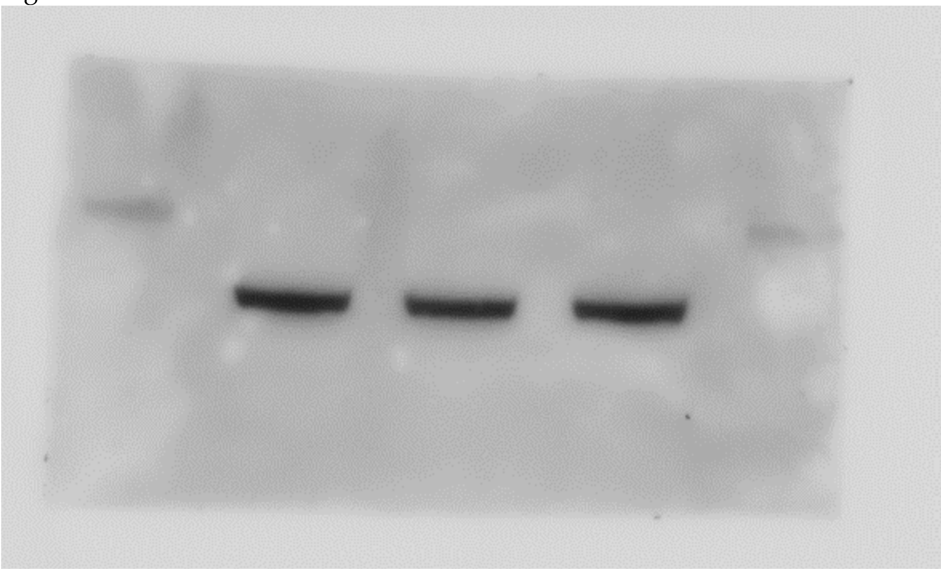

Figure 8C iNOS

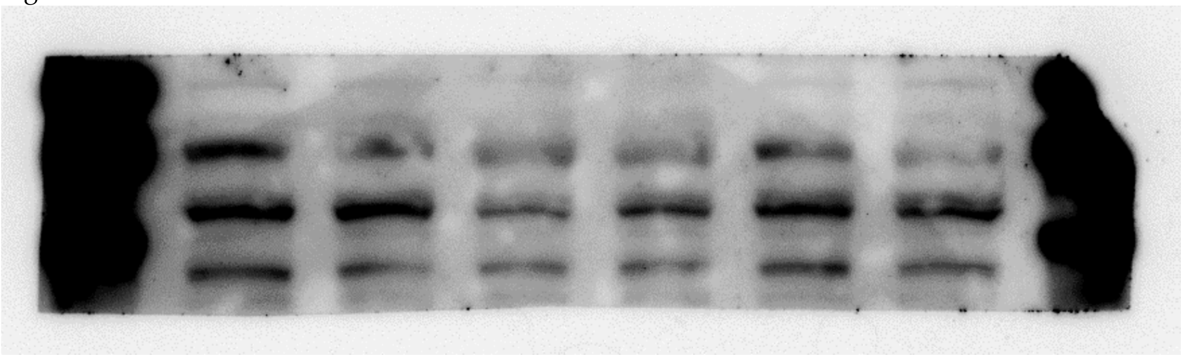

Figure 8C α-tubulin

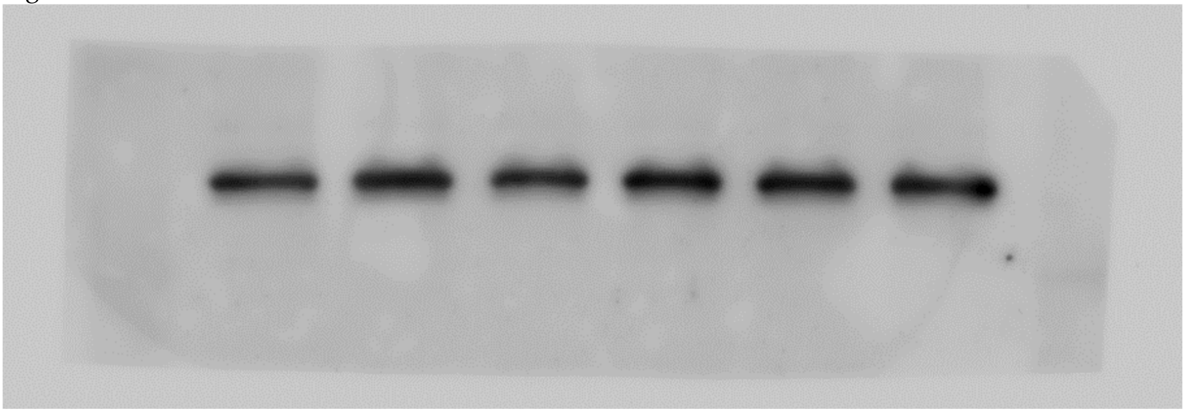

Figure 8D p-JNK

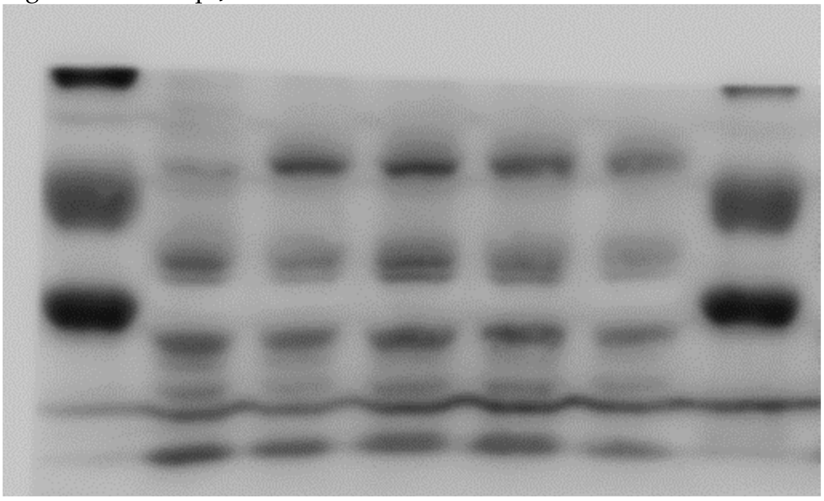

Figure 8D α-tubulin

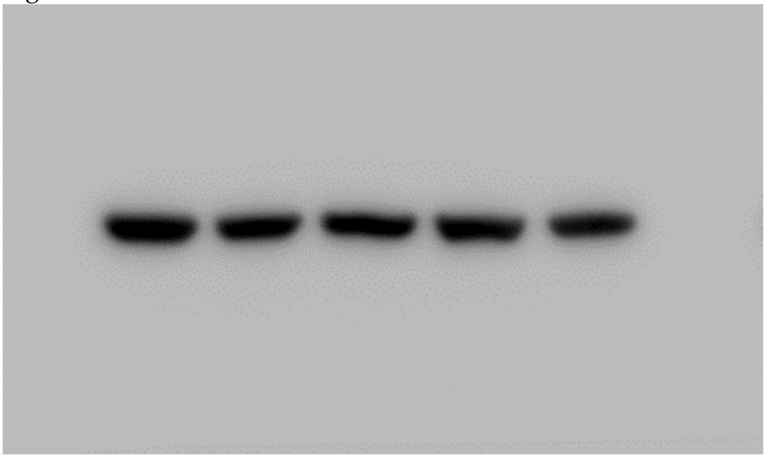

Figure 8E p-p38 MAPK

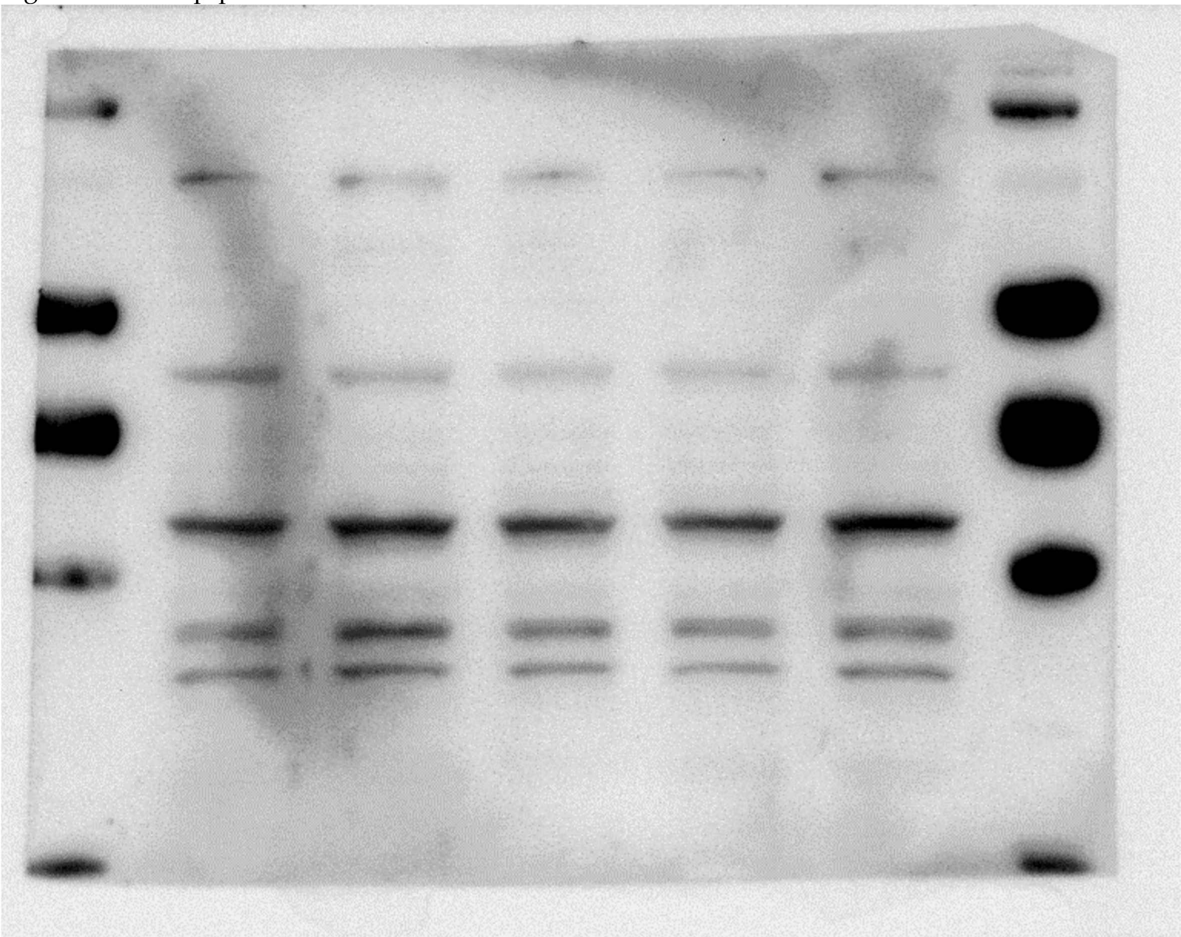

Figure 8E α-tubulin

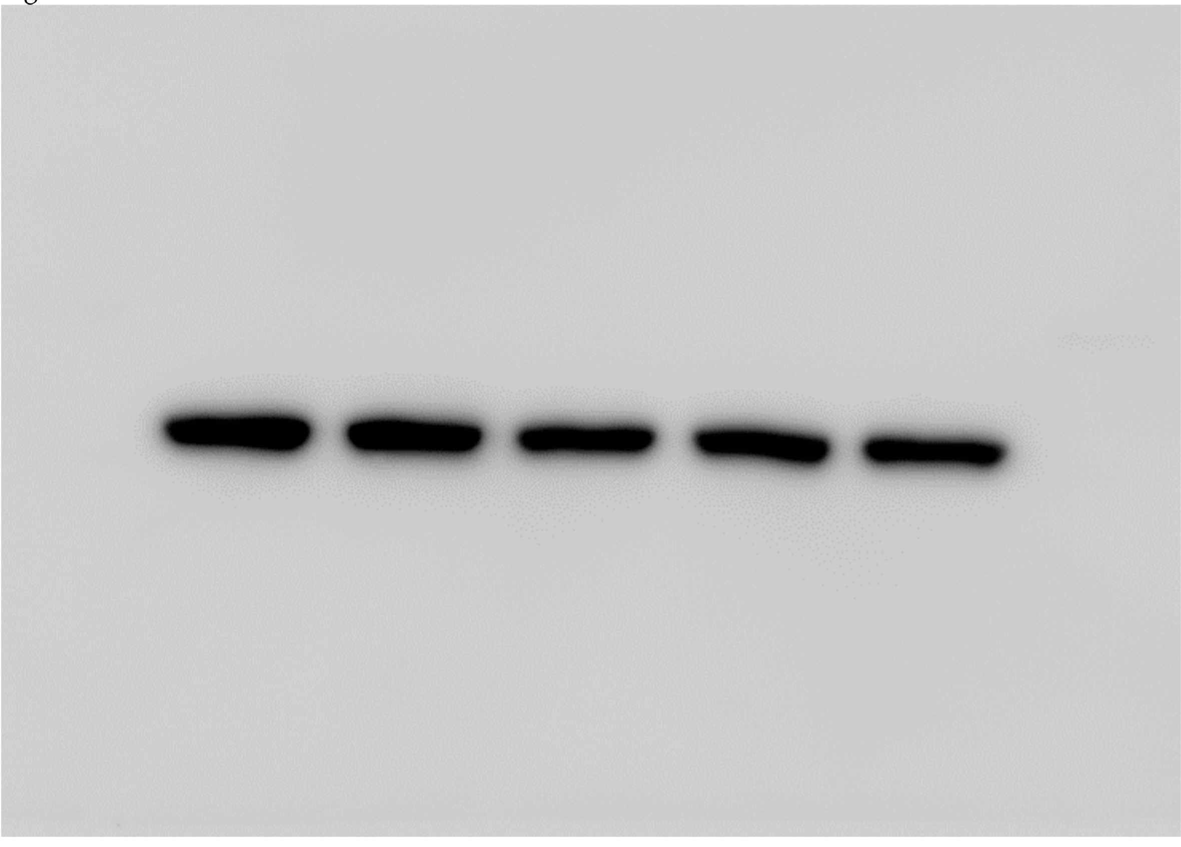

Supplement: Supplementary file 1 [file biomolecules-15-00174-s001.zip › File S1 original WB figures.pdf]
