# Supplementary figures and images for: Glabridin Suppresses Macrophage Activation by Lipoteichoic Acid In Vitro: The Crucial Role of MAPKs-IL-1β-iNOS Axis Signals in Peritoneal and Alveolar Macrophages
Source: Biomolecules. 2025 Jan 24;15(2):174. doi: 10.3390/biom15020174 (PMC11853366; doi:10.3390/biom15020174)

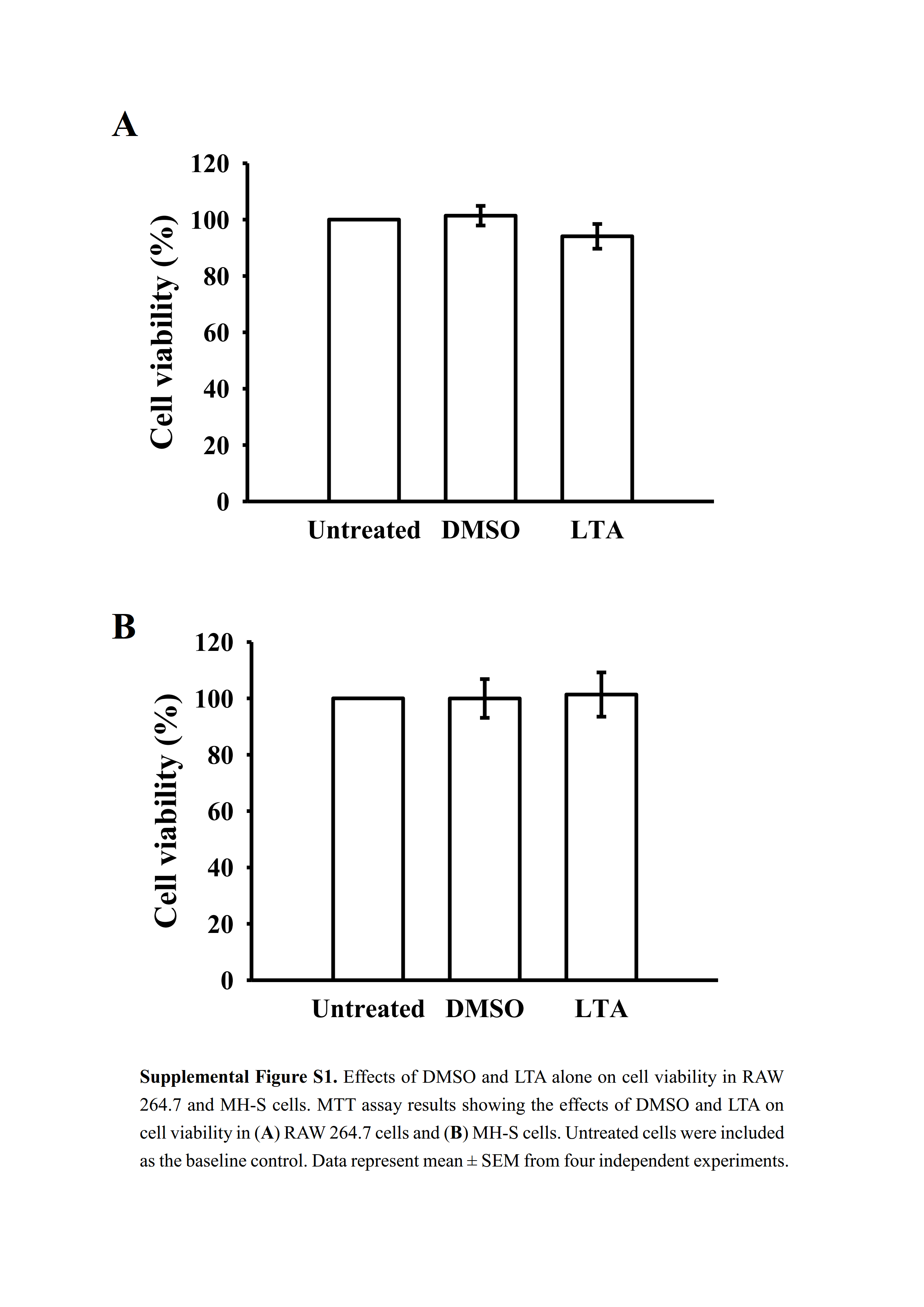

Supplement: Supplementary file 1 [file biomolecules-15-00174-s001.zip › biomolecules-3344553-supplementary Figure S1.tif]
